# Integrating multi-omic features exploiting Chromosome Conformation Capture data

**DOI:** 10.3389/fgene.2015.00040

**Published:** 2015-02-11

**Authors:** Ivan Merelli, Fabio Tordini, Maurizio Drocco, Marco Aldinucci, Pietro Liò, Luciano Milanesi

**Affiliations:** ^1^Bioinformatics Unit, Institute of Biomedical Technologies, Italian National Research CouncilMilan, Italy; ^2^Computer Science Department, University of TorinoTorino, Italy; ^3^Computer Laboratory, University of CambridgeCambridge, UK

**Keywords:** multi-omic data integration, Chromosome Conformation Capture, gene neighborhood map, chromatin spatial organization, linking gene regulatory elements

## Abstract

The representation, integration, and interpretation of omic data is a complex task, in particular considering the huge amount of information that is daily produced in molecular biology laboratories all around the world. The reason is that sequencing data regarding expression profiles, methylation patterns, and chromatin domains is difficult to harmonize in a systems biology view, since genome browsers only allow coordinate-based representations, discarding functional clusters created by the spatial conformation of the DNA in the nucleus. In this context, recent progresses in high throughput molecular biology techniques and bioinformatics have provided insights into chromatin interactions on a larger scale and offer a formidable support for the interpretation of multi-omic data. In particular, a novel sequencing technique called Chromosome Conformation Capture allows the analysis of the chromosome organization in the cell’s natural state. While performed genome wide, this technique is usually called Hi–C. Inspired by service applications such as Google Maps, we developed NuChart, an R package that integrates Hi–C data to describe the chromosomal neighborhood starting from the information about gene positions, with the possibility of mapping on the achieved graphs genomic features such as methylation patterns and histone modifications, along with expression profiles. In this paper we show the importance of the NuChart application for the integration of multi-omic data in a systems biology fashion, with particular interest in cytogenetic applications of these techniques. Moreover, we demonstrate how the integration of multi-omic data can provide useful information in understanding why genes are in certain specific positions inside the nucleus and how epigenetic patterns correlate with their expression.

## INTRODUCTION

What is the best way to integrate and represent omic data? This inquiry results critical in an era that is witnessing an explosion of the available molecular biology information. In particular, the integration and the interpretation of omic data in a systems biology view is complex, because actual representations rely on genomic coordinates, discarding at first gene spatial cooperation and renouncing to exploit the real conformation of the DNA in the nucleus. Moreover, approaches that are commonly used to annotate and analyze molecular biology experiments, such as ontology mapping and enrichment analysis, assume as prerequisite an independent sampling of features, which is clearly not satisfied while looking at long-range chromatin interactions (de Wit and de Laat, 2012), since they associate regions that are known to be functionally correlated.

Considering the number of experiments that highlight the importance of co-localization and co-expression of genes (Di Stefano et al., 2013), the possibility of mapping multi-omic features on a map capable of representing the effective disposition of genes in the nucleus can be of great utility. Moreover, the possibility of introducing network concepts to represent the behavior of genomic actors seems a suitable solution for the interpretation of this kind of data, since they allow to map a lot of information in complex, dynamical structures that organize items in an integrated way.

Recent advances in high throughput molecular biology techniques and bioinformatics have provided insights into chromatin interactions on a larger scale (Lieberman-Aiden et al., 2009). A novel technique called Chromosome Conformation Capture (3C) allows the analysis of chromosome organization in the cell’s natural state (Duan et al., 2012). The combination of high-throughput sequencing with this technique, generally called Hi–C, allows the characterization of long-range chromosomal interactions genome-wide (Lin et al., 2012). Hi–C gives information about coupled DNA fragments that are cross-linked together due to spatial proximity, providing data of the chromosomal arrangement in the 3D space of the nucleus. If used in combination with chromatin immunoprecipitation, 3C can be employed for the analyses of interactions between DNA and particular proteins, in a technique called ChIA-pet (Fullwood et al., 2009; Dixon et al., 2012; Li et al., 2012; Papantonis et al., 2012).

These techniques allow the description of the nucleus organization at unprecedented resolution, offering the possibility to study the structural properties and spatial organization of chromosomes. This is of critical importance for understanding and evaluating the regulation of gene expression, DNA replication, repair, and recombination (Chepelev et al., 2012). Moreover, using the Hi–C approach, the possibility of comparing the three-dimensional organization of the DNA in physiological and pathological conditions is achievable. The capability of describing how diseases reorganize the chromatin conformation to originate novel co-localized gene clusters of co-expression would be of primary importance.

To fully exploit the potential of this technique, many issues have to be faced. First of all the huge amount of data that should be produced for describing the conformation of the DNA in the nucleus. Considering that there are more than 200 different cell types with different profiles, which also change depending on the cell’s actual state, the sequencing effort required to describe the three-dimensional configuration of genes in the nucleus is huge. Moreover, the integration of epigenetic information that is strictly correlated to the DNA conformation in the cell in a mutual cross-regulation (since the expression of proteins that organize the chromatin in the nucleus is correlated to the conformation of the chromatin itself), making the data problem explosive.

In this paper we describe a initial attempt to analyze Hi–C data and related multi-omic features using a network approach to represent gene co-localization and co-regulation. In particular, we describe how the R package NuChart, with its algorithmic features that have been previously presented (Merelli et al., 2013), can be used to interpret 3C data for creating a map that represents multi-omic information. Here, we present the possibilities that can be opened by using systems biology concepts for the analysis of 3C data, in particular highlighting how this procedure has the potential to enter into clinical practice, because it provides information that can be interpreted in a cytogenetic view, with incomparable resolution and richness of details.

## MATERIALS AND METHODS

Inspired by web applications such as Google Maps, we developed NuChart (Merelli et al., 2013), an R package that elaborates Hi–C information to provide a systems biology oriented, gene-centric view of the three-dimensional organization of the DNA in the nucleus (the software, the manual, and all the supporting materials are freely available at ftp://fileserver.itb.cnr.it/nuchart). NuChart can be used to describe the DNA conformation in the neighborhood of selected genes by mapping on the achieved graph genomic features that are important for controlling gene expression at epigenetic level.

Although NuChart is the first R package allowing both visualization and analysis of Hi–C data in a gene-centric fashion [other software are CytoHi–C (Shavit and Lio’, 2013) and Homer (Heinz et al., 2010), which both rely on Cytoscape], a similar approach was initially presented by Wang et al. (2013), for the analysis of chromatin conformation data in experiments concerning acute lymphoblastic leukemia (ALL) and Lymphoma cells. This work pioneered the idea of analyzing the social behavior of genes by using a graph-based approach. A similar method has been exploited in NuChart, which in addition allows a statistical interpretation of both expression and epigenetic data in comparison to the topology of the graph, thus allows a deep integration of this kind of information.

For example, it is possible to map on the neighborhood graph Linking Gene Regulatory Elements [in particular, the predicted binding sites for the CTCF or Cohesin proteins (Botta et al., 2011)], isochores [that describe the variations in the GC content and are important for the genome organization (Varriale and Bernardi, 2009)], potential cryptic Recombination Signal Sequences [cRSSs, which are important for generating the antigen receptor diversity (Marculescu et al., 2006)], and other user desired genomic features (using the bed file format), such as methylation profiles and histone modifications, to infer how epigenetic features and the three-dimensional nuclear organization of DNA cooperate in controlling gene expression. This can be very useful while studying the differentiation of stem cells or for identifying chromosomal reorganizations in cancer cells.

The package is built upon the functionality of Bioconductor packages such as biomaRt, Biostrings, ArrayExpress, GEOquery, KEGGREST, limma, samr, igraph, and ergm, providing a novel method to exploit Hi–C data in a systems biology context. NuChart, used in combination with the Hicup software, processes Hi–C data in FASTQ format, performs some preliminary normalizations relying on the fragment distances from the enzymatic cut sites. The output is a detailed table concerning the chromosomal spatial neighborhood of the input genes, providing a related graph on which it is possible to map multi-omic features.

**FIGURE 1:**
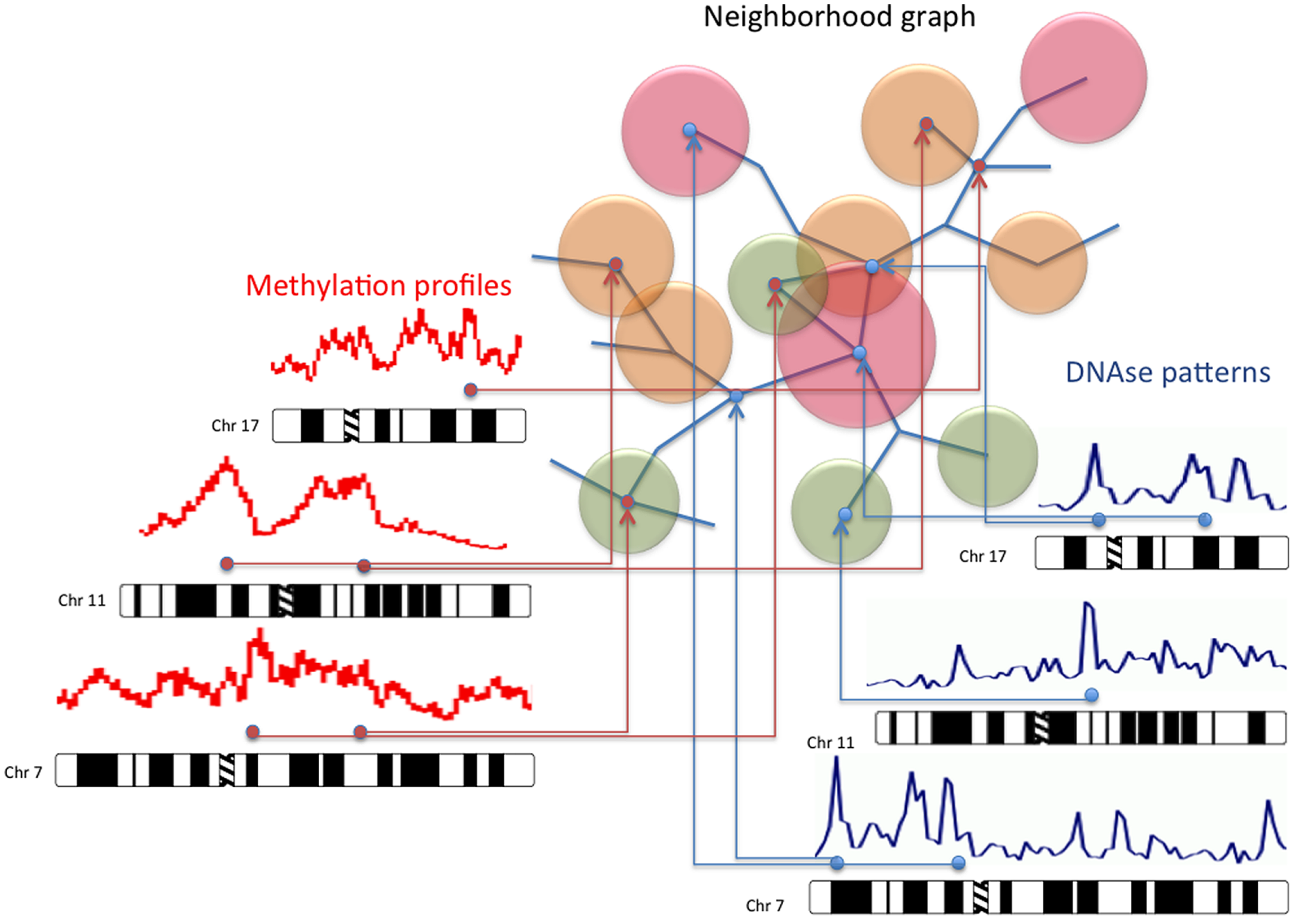
**Multi-omic integration of data using Hi**–**C maps.** The possibility of employing a map describing the chromatin organization in the nucleus to represent multi-omic data can be very useful for the interpretation of this kind of information. This approach recall the use of Google maps to display, on a cartographic representation of a city, features such as shops and places of interest. By using the same idea, we can map on the neighborhood graph of the genes inside the nucleus omic data such as methylation patterns, immunoprecipitation information, open-chromatin conformations, and expression profiles.

The idea behind this package is to provide a complete suite of tools for the analysis of Hi–C data using a gene-centric point of view to provide a map on which other omic data can be mapped (see **Figure 1**). Contact matrices, or better their probabilistic models, allow to create representations that only involve two chromosomes, while we are able to describe the interactions of all the chromosomes together using a graph-based approach. This representation gives more importance to the physical proximity of genes in the nucleus in comparison to coordinate-based representations. This is the same problem that impairs representations based on Circos, which are able to characterize the whole genome in one shot, but fail to describe the physical proximity of genes.

A typical analysis performed with NuChart starts with the pre-processing of the FASTQ file using Hicup, which provides as output a SAM file (see the Hicup documentation for more details). Then, data can be loaded into the R environment and normalized using a generalized linear model relying on a Poisson distribution (taking into account Hi–C fragment length, mappability and GC-content). Considering that this normalization approach is well-established (Hu et al., 2012), the algorithm returns the same results of other approaches relying on the computation of the contact matrices (Servant et al., 2012; Seitan et al., 2013; Ay et al., 2014), providing a probability score at each edge of the neighborhood graph.

This method allows to estimate, at the same way of the contact maps, the probability that different genomic regions are proximal one to the other, with the advantage of allowing an iterative analysis of the space: it is therefore possible to calculate the probability that two genes are distant a specific number of contacts. Moreover, the graph-based description of gene positions in the nucleus is extremely useful for mapping other multi-omic features, since analyzing data through this spatial description of the DNA conformation allows the identification of long-range interactions, cooperative genes and common epigenetic patterns, which are more difficult to identify relying on chromosomal coordinates.

The core procedure starts from one or more input genes from which a graph of adjacent genes is constructed. The identification of neighbor genes begins searching chromosome fragments that belong to the input genes. These fragments are then compared with other chromosome fragments located in a different genomic region, as reported by coupled reads. When a match is found and a new fragment is identified within a specific gene region, an edge between the starting gene and the novel detected one is created. A very important feature of the algorithm is the possibility to specify the number of iterations to accomplish for creating the neighborhood graph, which means to specify the maximum span that the graph can reach starting from the input genes (correlated to the diameter of the graph or, using the graph theory terminology, to the “longest shortest path”).

By default this value is set to one, which means that, considering the list of genes given as input and taking into account the desired normalization, only genes that are directly in contact are mapped on the graph. If this parameter is set to two the procedure is iterated twice, meaning that all the genes identified in the neighborhood of the input genes at the first iteration of the algorithm are searched again for Hi–C interactions with other genes. And so on. This is of critical importance because it allows to overcome the limit of the contact matrix representation, which is limited by definition at representing only the interactions just one step away from the considered gene, while here we can identify paths also between distant genes.

The added value of this package is to provide the possibility of analyzing Hi–C data in a multi-omic context, by enabling the capability of mapping on the graph vertices expression data, according to a particular transcriptomic experiment, and on the edges genomic features that are known to be involved in chromosomal recombination, looping, and stability. If the user is interested in mapping on the neighborhood graph also gene expression data, there are functions for downloading microarray experiment results from ArrayExpress and GEO. Moreover, using NuChart it is possible to map on the neighborhood graph many genomic features such as data concerning cryptic RSSs, isochores, and CTCF binding sites, which are embedded in the package, but also any other omic information using the common bed file format.

NuChart also provides three functions to describe, compare, and statistically analyze neighborhood graphs once they have been created, which can be useful to highlight local and global characteristics of the fragment distribution in the context of the three-dimensional DNA topology inside the nucleus. In particular, there is the possibility to create general statistics about the graphs, which can be useful to describe physiological and pathological conditions of the cells, verifying the differences in the spatial distribution of genes. Then, neighborhood graphs can be compared by applying a conversion in adjacency matrices and then employing the Pearson correlation to check their similarity (for example to see intra and inter experiments variability).

The last set of functions available in NuChart enables the user to analyze, from a statistical point of view, the neighborhood graphs in relation to the mapped multi-omic features. In particular, these functions rely on the R package Exponential-family Random Graph Models (ERGMs) that provides an integrated set of tools for creating an estimator of the network through a stochastic modeling approach. In particular, the ERGM functions are able to extrapolate the salient characteristics of a network by implementing a maximum likelihood estimator.

Operatively, the software generates a huge number of networks, selects the ones having characteristics similar to the graph under analysis (i.e., degree distribution, connected components, topological conformation), and tries iteratively to optimize the generation parameters until all the created graphs have characteristics similar to those processed. This estimator is extremely useful, since it allows to create a probability distribution by which some peculiarities of the graph can be extrapolated, concerning both its intrinsic topology and specific attributes of the nodes (Admiraal and Handcock, 2007). In particular, the package allows to compute simple statistics about the topology of the graph, such as the significance of the vertex clustering attitude (triangle), or the significance of the network tendency to create multiple paths between two vertices (twopath). On the other hand, by choosing more complex modeling functions and exploiting the mapped multi-omic features, NuChart allows to test, for example, the probabilities that edges are a function of a specific genomic feature (nodecov) or the significance of having edges in relation to the absolute difference of a vertices’ property (absdiff). The possibility of analyzing data to infer structural-activity relationships in a network is of critical importance (Reagans and McEvily, 2003).

## RESULTS AND DISCUSSION

In this section we present some applications of the NuChart package. In particular, we show some interesting results relying on the possibility of creating metrics for defining how far two genes are one from the other, with possible applications to cytogenetic profiling, to the analysis of the DNA conformation in the proximity of the nucleolus, and for describing the social behavior of genes.

### APPLICATION TO CYTOGENETIC

Applications of 3C techniques to cytogenetics are becoming very appealing, because the relative position of genes can be identified using high-throughput experiments. An example can be found in the work of Naumova et al. (2013), which concerns the analysis of the mitotic chromosome organization, while other studies showed how it is possible to identify translocations in Hi–C data (Rusk, 2014). Here we show how Hi–C can be used for diseased versus normal cells comparisons, with particular interest in leukemias, since it reproduces results achieved by Fluorescence *in situ* hybridization (FISH) experiments.

Although Hi–C is intended to estimate the contact frequencies between different genomic regions, there is a clear correlation with chromosomal translocations, since recombinations are largely influenced by the distance between fragments in which DNA breaks, necessary for translocations, occur. There are already many evidences in this sense (Meaburn et al., 2007; Engreitz et al., 2012; Shugay et al., 2012; Zhang et al., 2012; Kenter et al., 2013), which demonstrate how the physical distance plays a leading role for recombinations, in particular when the frequency of DNA breaks are physiological (while in cellular models where a high number of translocation are artificially induced the frequency becomes the dominant factor). Considering the association between contact frequencies and translocations, we think that a graph-based approach may be useful for data analysis from a recombination point of view. NuChart is capable of providing an immediate representation of genomic segments that are more likely to translocate with a specific gene, taking into account that the recombination probability is proportional to the weight of the connecting edges, according to the employed normalization.

The first example we present concerns the Philadelphia translocation, which is a specific chromosomal abnormality associated with chronic myelogenous leukemia (CML). The presence of this translocation is a highly sensitive test for CML, since 95% of people with CML have this abnormality, although occasionally it may occur in acute myelogenous leukemia (AML). The result of this translocation is that a fusion gene created from the juxtaposition of the ABL1 gene on chromosome 9 (region q34) to part of the BCR (“breakpoint cluster region”) gene on chromosome 22 (region q11). This is a reciprocal translocation, creating an elongated chromosome 9 (called der 9), and a truncated chromosome 22 (called the Philadelphia chromosome).

Using NuChart we compared the distance of some couples of genes that are known to create translocation in CML/AML. In particular, our analysis relies on data from the experiments of Lieberman-Aiden et al. (2009), which consist in four lines of karyotypically normal human lymphoblastoid cell line (GM06990) sequenced with Illumina Genome Analyzer, compared with two lines of K562 cells, an erythroleukemia cell line with an aberrant karyotype. Starting from well-established data related to the cytogenetic experiments (Dewald, 2002), we tried to understand if the Hi–C technology, in combination with NuChart, can successfully be applied in this context, by verifying if translocations normally identified by using FISH can also be studied using 3C data. Therefore, we identified five couples of genes that are know to be involved in translocations and we compared their Hi–C interactions in physiological and diseased cells.

**FIGURE 2:**
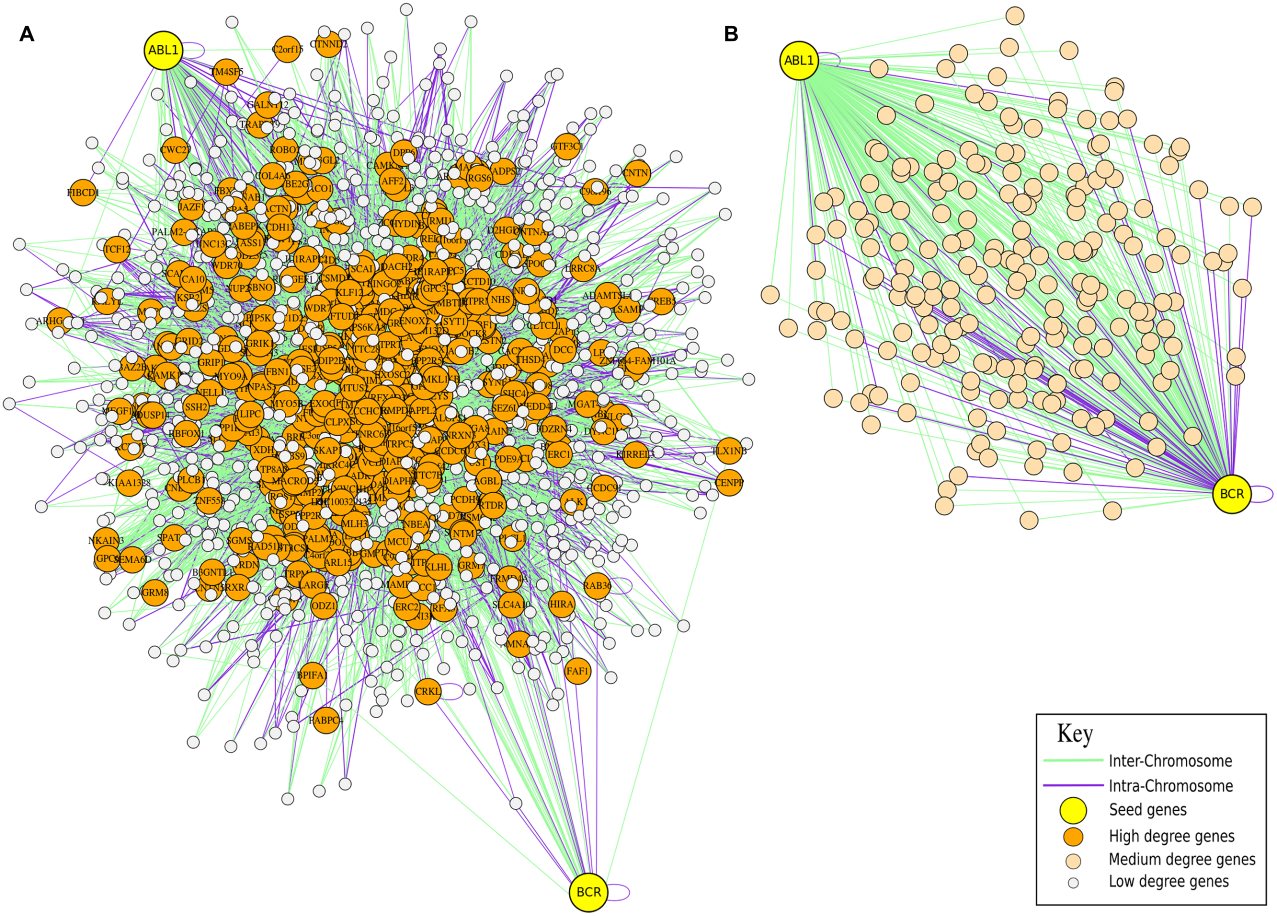
**Hi**–**C for Cytogenetic application.** Comparison of FISH data and Hi**–**C information for the cytogenetic analysis of translocation in leukemia. In panel **(A)** the neighborhood graph of the genes ABL1 and BCR generated from the GM06990 cell line (Lieberman-Aiden experiment SRA:SRR027956) is presented. The graph shows that in normal cells the genes are not directly in contact since other genes are placed between them. In panel **(B)** the neighborhood graph of the genes ABL1 and BCR from the K562 cell line (Lieberman-Aiden experiment SRA:SRR027962) is presented. The graph shows that in cancer cells the genes are closer one to the other, which is a preliminary evidence of a possible translocation.

The very interesting result is that ABL1 and BCR, considered a normalization equivalent to the one achieved with HicNorm, are likely to be distant 1 or 2 contacts (*p* < 0.05) in sequencing runs concerning GM06990 with HindIII as digestion enzyme (SRA:SRR027956, SRA:SRR027957, SRA:SRR027958, SRA:SRR027959), while they are directly in contact (*p* < 0.05) in sequencing runs related to K562 with digestion enzyme HindIII (SRA:SRR027962 and SRA:SRR027963). Therefore, there is a perfect agreement between the positive and the negative presence of Hi–C interactions and FISH data (see **Figure 2**). At the same way, AML1 and ETO are in close proximity (*p* < 0.05) in leukemia cells (SRA:SRR027962 and SRA:SRR027963), while they are likely to be far 2 or 3 contacts (*p* < 0.05) in normal cells (SRA:SRR027956, SRA:SRR027957, SRA:SRR027958, SRA:SRR027959). Considering the translocation CBFβ-MYH11, these genes are distant 2 or 3 contacts (*p* < 0.05) in GM06990 (SRA:SRR027956, SRA:SRR027957, SRA:SRR027958, SRA:SRR027959), while they are proximal with high probability (*p* < 0.05) in K562 (SRA:SRR027962, but not in SRA:SRR027963). We had no significant results for NUP214-DEK and PML-RARα translocations, which, however, are more rare in this kind of disease.

A second example of Hi–C cytogenetic application concerns the experiments of Wang et al. (2013) about B-cell ALL. Also in this disease there are well-characterized translocations, the most important of which is the TEL-AML1 fusion gene (Stams et al., 2005) that is present in about 25% of patients. This translocation of chromosome 12 (region q34) and chromosome 21 (region q22) results in the expression of chimeric transcription factors, which block both differentiation and apoptosis by interfering with the function of their wild-type counterparts.

As before, we employed NuChart to characterize the distance between some couples of genes in the cells’ physiological and pathological state. In detail, we used the results of the 4 karyotypically normal human lymphoblastoid cell line (GM06990) from the experiments of Lieberman-Aiden as control data (as in the Wang’s paper), while pathological profiles are directly taken from the experiments performed by Wang et al. (2013) (private communication). This dataset consists of 2 highly overlapping Hi–C experiments, the first concerning a case of primary human B-Cell ALL (B-ALL) and the second regarding the MHH-CALL-4 B-Cell ALL cell line (CALL4). Also in this case, starting from some well-established translocations, we tested the capability of the Hi–C technique, in combination with NuChart, to capture some genomic rearrangements usually identified using FISH.

The first result is that TEL and AML1, considered a normalization equivalent to the one achieved with HicNorm, are always distant 2 contacts (*p* < 0.05) in sequencing runs concerning GM06990 with HindIII as digestion enzyme (SRA:SRR027956, SRA:SRR027957, SRA:SRR027958, SRA:SRR027959), while they are directly in contact (*p* < 0.05) in sequencing runs related to B-ALL and CALL4. Other tests were performed on the E2A-PBX translocation: these genes are in close proximity (*p* < 0.05) in cancer cells (B-ALL and CALL4), while they are likely to be far 2 or 3 contacts (*p* < 0.05) in three out four control cell lines (SRA:SRR027956, SRA:SRR027958, SRA:SRR027959). Following the results discussed in the work of Taylor et al. (2013) we also tested the proximity of genes IGH and miR125b1 (related to a microRNA), which are distant 2 or 3 contacts (*p* < 0.05) in GM06990 (SRA:SRR027956, SRA:SRR027958, SRA:SRR027959), while they are proximal with high probability (*p* < 0.05) in leukemias cells (CALL4, but not in B-ALL, which presents a lower reads density). Considering the translocation BCR–ABL1, genes are distant 2 or 3 contacts (*p* < 0.05) in GM06990 (SRA:SRR027956, SRA:SRR027957, SRA:SRR027958, SRA:SRR027959), while they are proximal with high probability (*p* < 0.05) in leukemias cells (CALL4, but not in B-ALL, which presents a lower reads density). We had no results for the MLL and AF4 translocation.

These results are of significant importance, because with the decreasing of sequencing costs the Hi–C technique can be an effective diagnostic option for cytogenetic analysis, with the possibility of improving the knowledge regarding the correlation between the genome architecture and translocations. For example, Hi–C can be used to infer non trivial risk markers related to aberrant chromosomal conformation, like the Msc5a loci for breast cancer, which is known to play a critical role in the re-organization of a portion of chromosome 9 by CTCF proteins.

### RNA POLYMERASES

In the following example, we discuss an interesting analysis regarding the internal organization of the DNA in the nucleus, working on the data produced in the Dixon et al. (2012) experiments. The intention is to show the different chromosomal organizations that occur in the nucleolus, while gene expression is heavily characterizing the differentiation of stem cells, since this part of the nucleus is involved in the transcription of ribosomal RNA (rRNA) subunits and in their combination with proteins to form complete ribosomes. Therefore, at the border of the nucleolus are exposed transcriptional units ready to express genes, and it would be very useful to understand the organization of these structures in relation to genomic regions that are going to be transcribed.

For this reason, we performed an Hi–C analysis of some specific subunits of the RNA Polymerase I (that only transcribes rRNA), RNA Polymerase II (directly involved in microRNA and gene expression), and RNA Polymerase III (mainly required to express tRNA) to shed light in their different configurations in different cell types. While most of the subunits are shared, some are peculiar of a particular RNA Polymerase and we choose to use these subunits to verify if there is correlation between their position in the nucleus and their activities. Respectively, the neighborhood graphs have been produced according to two different sequencing runs performed on human embryonic stem cells (SRA:SRR400260 and SRA:SRR400261), and from human lung embryonic fibroblast (SRA:SRR400266 and SRA:SRR400267) of Dixon et al. (2012) experiments.

**FIGURE 3:**
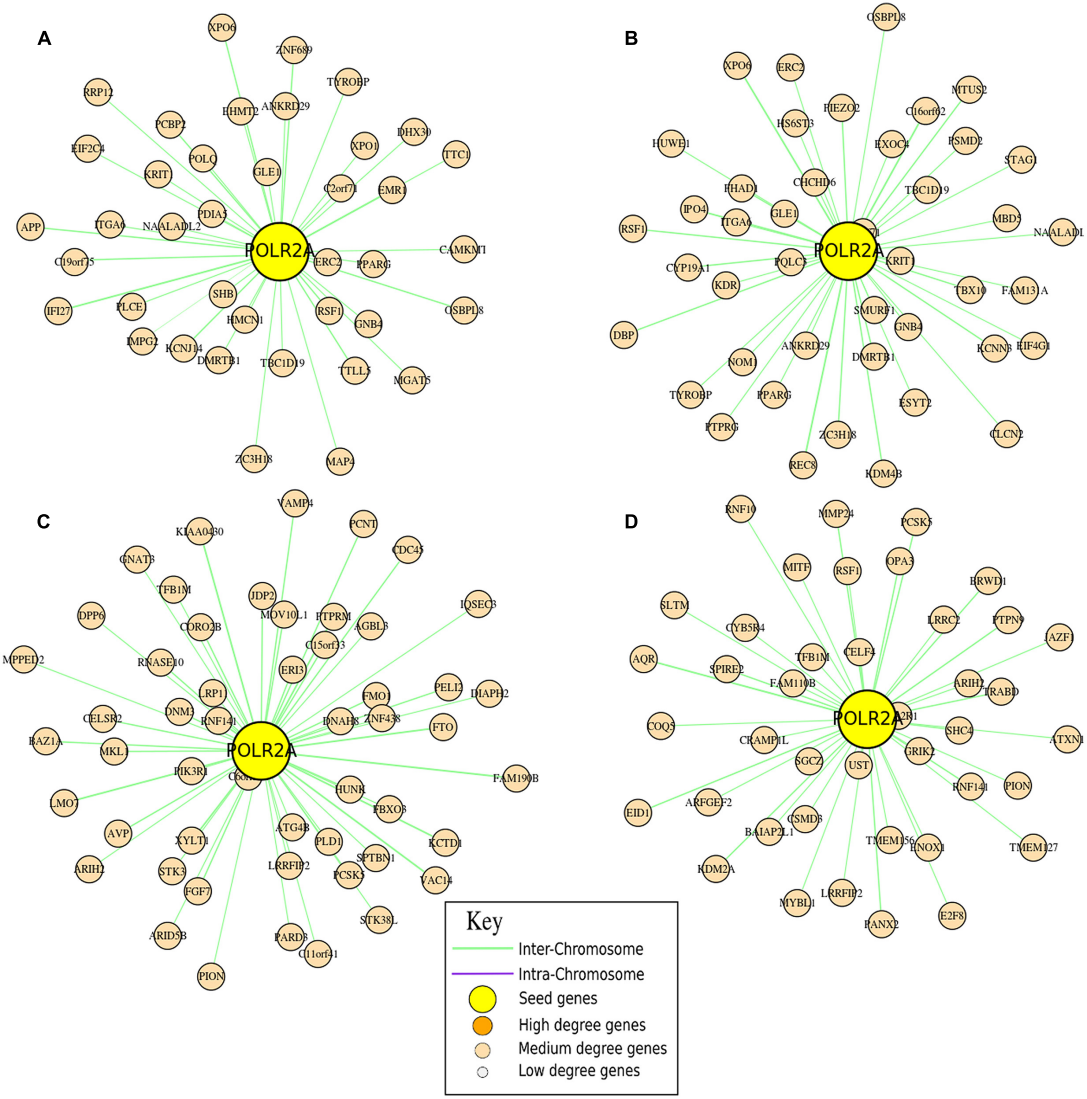
**Analysis of the nucleolus.** Neighborhood graphs of the gene POLR2A in four different runs from the Hi**–**C experiments of Dixon et al. (2012) to show inter and intra run modifications of the chromatin conformation. Panels **(A,B)** concern sequencing runs from hESC (SRA:SRR400260, SRA:SRR400261), while data in panels **(C,D)** are from IMR90 (SRA: SRR400266, SRA:SRR400267). In these graphs, seed genes are the genes given as input to the algorithm, while output genes are differentially represented according to their importance (in terms of degree).

In **Figure 3** a detailed representation of the different RNA Polymerase II neighborhood graphs is shown. In particular, these graphs show the neighborhood of the POLR2A gene that encodes for RPB1 (Strachan and Read, 1999), the largest subunit of the RNA polymerase II, which catalyzes the transcription of DNA to synthesize precursors of mRNA, most snRNA and microRNA, in the different cell lines. The representation shows that there are a wide range of genes involved in cell differentiation, with an enrichment of genes related to the cell cycle process (such as CDC45 and CCNE1, CCNB1) and many transcription factors (such as EBF1, TFEC, TFAP2A, TFB1M). Concerning POLR1A, that encodes for the A190 protein of the RNA Polymerase I, in the different experiments, as expected, it has in its neighborhood genes that are correlated to the rRNA subunits, such as RPL31, MPRS5, MRPS9, MRPS24, MRPS27, and MRPL35. Regarding POLR3B, which encodes for the subunit C128 of the RNA Polymerase III, we found in its neighborhood a couple of genes related to tRNA, in particular TRNAD1 (transfer RNA aspartic acid 1 – anticodon GUC) and TRNAS26 (transfer RNA serine 26 – anticodon AGA).

Considering the variability in the neighborhood of these genes, computed as correlation between lists of adjacent genes, there is a wide changeability looking at the RNA Polymerase II, while the differences considering RNA Polymerase I and III are considerably smaller. In particular, the similarity between two different runs of sequencing performed on the same cell type is relatively high for DNA Polymerase II (respectively, 60 and 67%), while there are very important differences between the two cell lines (correlation below 30%), which witnesses the importance (and the variability) that chromosomal re-organizations have at the nucleus/nucleolus level for co-expression. Considering DNA Polymerase I and III, there is a high reproducibility for runs performed on the same samples (respectively, 85 and 87% for POLR1A and 80 and 83% for POLR3B) and a relative increase in the analyses performed in different cell lines (correlation around the 40%). This kind of analysis is very important for understanding, in a particular moment, what the cells are going to express by reorganizing their chromosomal structure in the three-dimensional space of the nucleus.

### NETWORK MODELING

**FIGURE 4:**
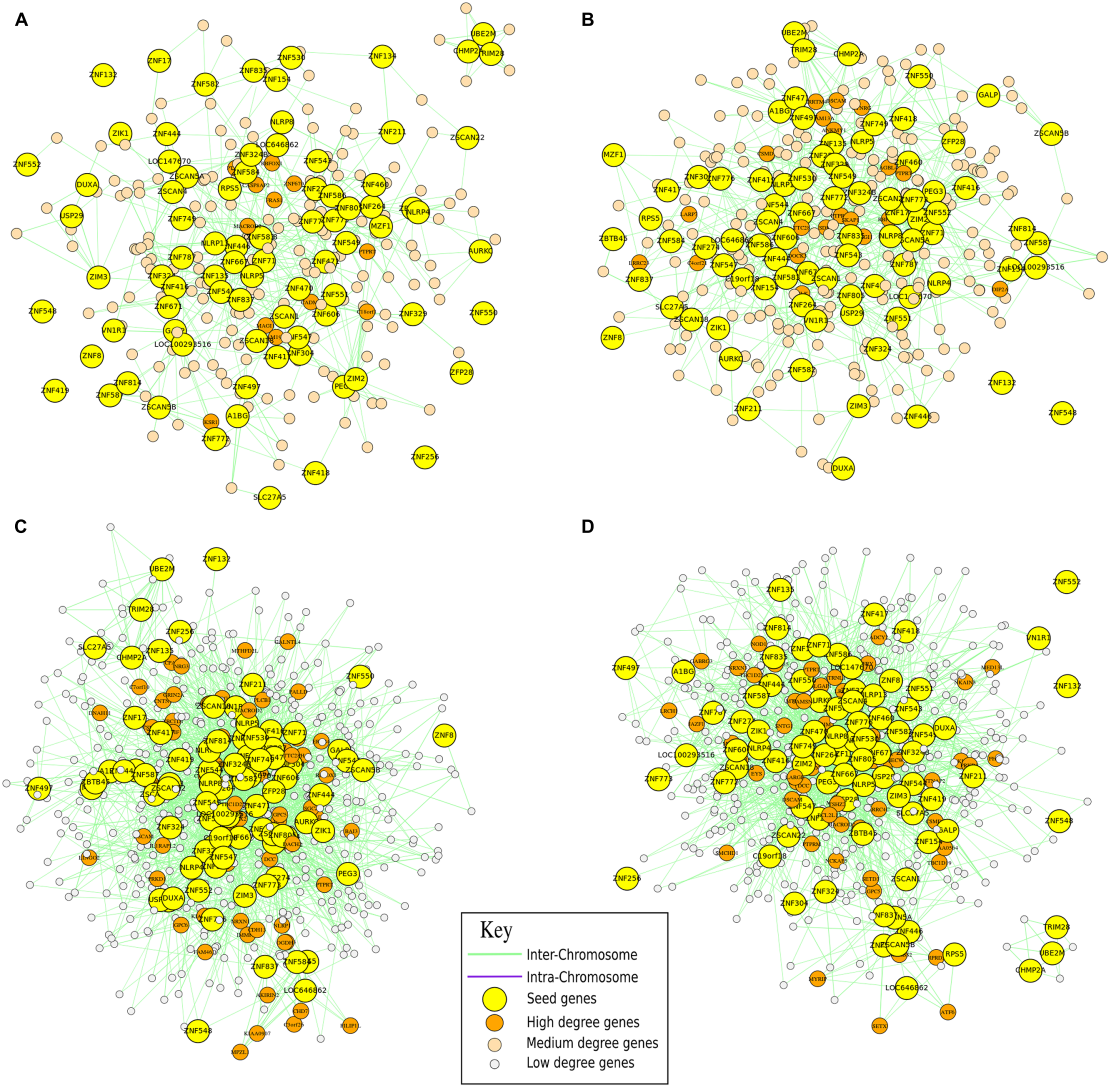
**Gene clustering analysis.** Neighborhood graphs of the Kruppel-type zinc finger cluster of genes (cytoband chr19.q13.12), related to the Kruppel-associated box (KRAB), in four different runs from the Hi**–**C experiments of Dixon et al. (2012) to show inter and intra run modifications of the chromatin conformation. Panels **(A,B)** concern sequencing runs from hESC (SRA:SRR400260, SRA:SRR400261), while data in panels **(C,D)** are from IMR90 (SRA: SRR400266, SRA:SRR400267). In these graphs, seed genes are the genes given as input to the algorithm, while output genes are differentially represented according to their importance (in terms of degree).

**FIGURE 5:**
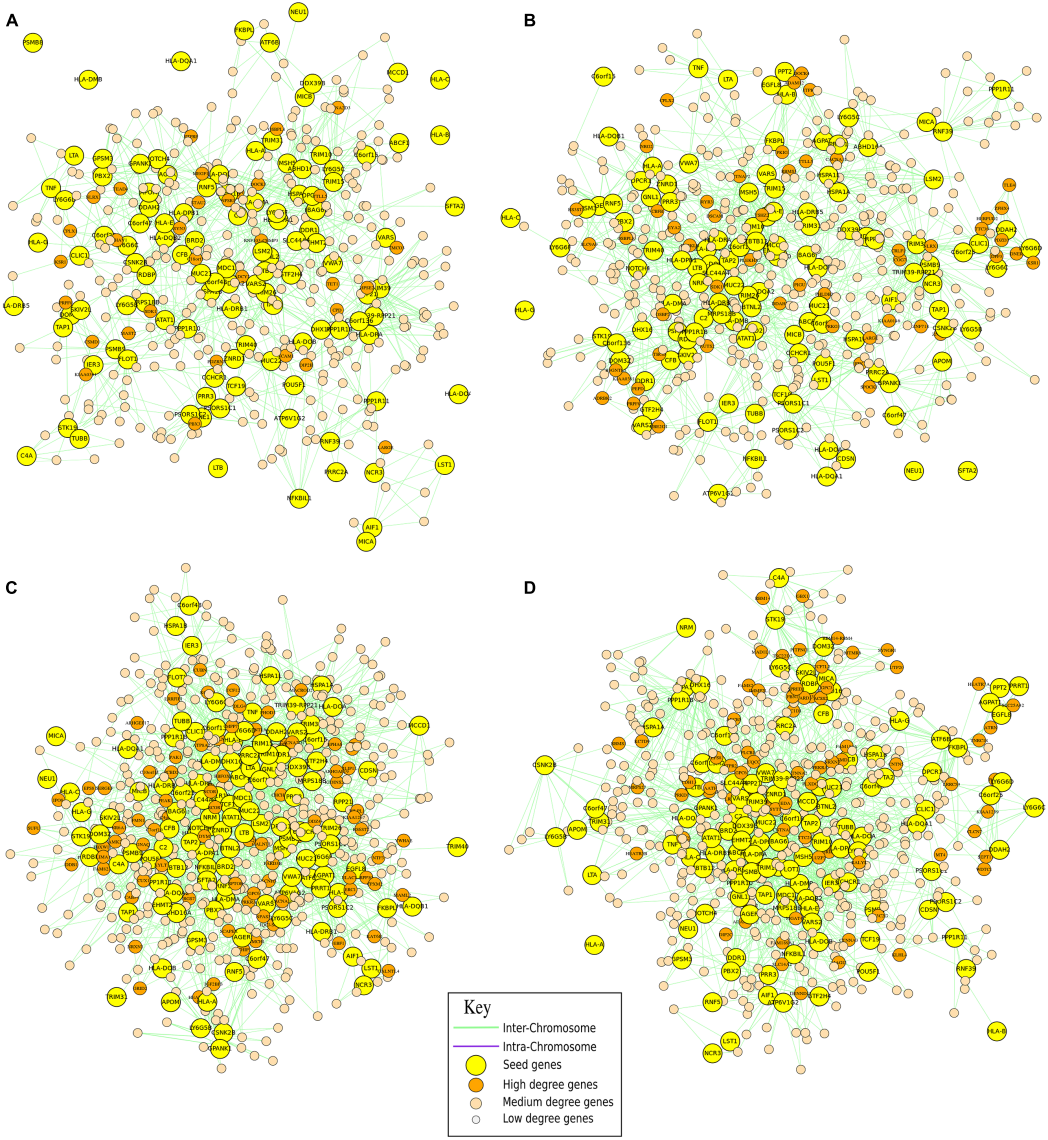
**Gene clustering analysis.** Neighborhood graphs of the human leukocyte antigen (HLA) cluster of genes (cytoband chr6.p31.21) in four different runs from the Hi–C experiments of Dixon et al. (2012) to show inter and intra run modifications of the chromatin conformation. Panels **(A,B)** concern sequencing runs from hESC (SRA:SRR400260, SRA:SRR400261), while data in **(C,D)** are from IMR90 (SRA: SRR400266, SRA:SRR400267). In these graphs, seed genes are the genes given as input to the algorithm, while output genes are differentially represented according to their importance (in terms of degree).

The power of NuChart relies on the capability of capturing and describing the co-localization and co-activation of single entities in a gene network, exploiting a systems biology approach. Moreover, the interaction of the actor genes with the environment is of critical importance for understanding the entire system. This can be performed using the modeling functions of the package, which allow to statistically characterize the distribution of the edges in relation to the characteristics of the nodes that are the mapped multi-omic features. In order to show the possibilities of NuChart in terms of statistical inference on the graph, we performed the analysis of the clusters of genes Kruppel-associated box (KRAB; **Figure 4**) and human leukocyte antigen (HLA; **Figure 5**) in the context of four Dixon et al. (2012) experiments to verify the correlation of the edge distribution in relation to some genomic features (hypersensitive sites, CTCF binding sites, isochores, RSSs), whose data are embedded in the NuChart package.

The first analyzed locus is located in cytoband chr19.q13.12 and concerns the clusters of Kruppel-type zinc finger genes, related to the KRAB, that are distinctive for their tandem organization (Huntley et al., 2006). Zinc finger proteins are a family of transcription factors that regulate the gene expression, and most of these proteins are members of the KZNF family. There are seven human-specific novel KZNFs and 10 KZNFs that have undergone pseudo-gene transformation specifically in the human lineage. 30 additional KZNFs have experienced human-specific sequence changes that are presumed to be of functional significance. Members of the KZNF family are often in regions of segmental duplications, and multiple KZNFs have undergone human-specific duplications and inversions.

The second analyzed gene cluster concerns the HLA system, which is the name of the locus containing the genes that encode for major histocompatibility complex (MHC) in humans. The proteins encoded by these genes are also known as antigens, as a result of their historic discovery as factors in organ transplants. The HLA belongs to a super-locus that contains a large number of genes related to the immune system function in humans. In particular, this group of genes resides on cytoband chr6.p31.21 and encodes for cell-surface antigen-presenting proteins, which have many different functions. Primarily, the HLA complex helps the immune system distinguish the body’s own proteins from proteins made by foreign invaders such as viruses and bacteria.

These statistical results are quite intriguing to analyze (**Table 1**). From one side, the correlation between the presence of CTCF binding sites and edges was predictable since Linking Gene Regulatory Elements are demanded to keep different regions of the genome close to each other, but is very interesting to quantify this association. On the other hand, regions with isochores seem less involved in long-range interactions, which can be quite surprising considering that these portions of the genome are considered gene-rich. The correlation between cryptic RSS sites and edges is more pronounced in the HLA cluster in comparison to the KRAB cluster, probably due to a more consistent presence of this kind of sequences in genes related to the immune system. Finally, the correlation between hypersensitive sites (super sensitivity to cleavage by DNase) and edges, although positive, is poor, probably because the accessibility of these regions are impaired by a large number of long-range interactions.

**Table 1:** Analyses of CTCF binding sites, isochores, cryptic RSSs, and hypersensitive sites (super sensitivity to cleavage by DNase) impact on the edge distribution of the KRAB cluster of genes and of the HLA cluster of genes.

	KRAB		HLA	
	Estimate	SE	Estimate	SE
**SRA:SRR400260**
Edges + nodecov(“dnase”)	0.2867	0.08451	0.1751	0.07961
Edges + nodecov(“ctcf”)	0.6531	0.01157	0.5845	0.01253
Edges + nodecov(“rss”)	0.5804	0.06176	0.6304	0.08196
Edges + nodecov(“iso”)	-1.0470	0.09269	-0.9406	0.09156
**SRA:SRR400261**
Edges + nodecov(“dnase”)	0.2042	0.06782	0.1706	0.08022
Edges + nodecov(“ctcf”)	0.6629	0.04158	0.6287	0.03225
Edges + nodecov(“rss”)	0.5378	0.03566	0.6419	0.03776
Edges + nodecov(“iso”)	-1.0151	0.09566	-0.9335	0.08969
**SRA:SRR400266**
Edges + nodecov(“dnase”)	0.3042	0.05962	0.1818	0.07822
Edges + nodecov(“ctcf”)	0.6738	0.03744	0.5678	0.02113
Edges + nodecov(“rss”)	0.5569	0.02996	0.6617	0.03776
Edges + nodecov(“iso”)	-1.1000	0.09655	-0.8305	0.08969
**SRA:SRR400267**
Edges + nodecov(“dnase”)	0.3272	0.07932	0.1901	0.05925
Edges + nodecov(“ctcf”)	0.6645	0.04158	0.4677	0.02005
Edges + nodecov(“rss”)	0.5378	0.02755	0.6520	0.03883
Edges + nodecov(“iso”)	-0.9501	0.09076	-0.8707	0.09050

*SE, Standard Error.*

*It’s very interesting to highlight the high similarities between the four sequencing runs. In particular, data demonstrates that CTCF binding sites and cryptic RSSs have a positive influence on the presence of edges. At the same way DNase hypersensitive sites are positively correlated with edges although with less impact, while isochores are negatively correlated with the edge distribution.*

## CONCLUSION

The integration and visualization of omic data is a critical issue and they really represent challenges for scientists that work on Big Data paradigms in the 21st century. Tools to integrate a cascade of multi-omic data with the information about the structure of the nucleus require a cartographic approach such as Google maps, because genome browsers only work at the coordinate level, discarding long-range interactions and associations.

Changing the point of view into a more systems biology fashion, we think that the information about the chromatin organization may also be the key to interpret this multi-omic cascade of data, since they are capable of providing genetic maps to make clearer the collective behavior of genes. The cooperation among genes can probably be better interpreted using tools that are typical of the social network era and the possibility to use tools like NuChart supports this concept. In particular, the possibility of having suitable descriptions of how genes are localized in the nucleus, enriched by genomic features that can characterize the way they are capable of interacting, and combined with statistical analysis and semantic tools may result extremely useful in the years to come.

The interpretation of epigenetic features, genomic patterns, DNA binding sites, co-expression patterns could take an incredible advantage from the availability of distance matrices between genes, which can provide a measure of their correlation. Vice versa, due to the close connection between the three-dimensional organization of the DNA in the nucleus and the multi-omic features that regulate the cellular machinery, distance information can provide new hints about clusters of genes that cooperate under the control of the same transcription factors for specific biological processes.

## Conflict of Interest Statement

The authors declare that the research was conducted in the absence of any commercial or financial relationships that could be construed as a potential conflict of interest.
